# Is the pursuit of happiness the pursuit of homeostasis? A review on the modulatory functions of endorphins on human behavior

**DOI:** 10.1192/j.eurpsy.2022.1893

**Published:** 2022-09-01

**Authors:** M. Conde Moreno, F. Ramalheira

**Affiliations:** 1 Centro hospitalar Psiquiátrico de Lisboa, Hospital De Dia, Lisboa, Portugal; 2 Centro hospitalar Psiquiátrico de Lisboa, Serviço De Electroconvulsoterapia, Lisboa, Portugal

**Keywords:** endorphins, endogenous opioids, human behavior

## Abstract

**Introduction:**

Endorphins have been associated with analgesia and pleasurable activities. However, the so-called “happy chemicals” are far more complex than initially thought. Research shows that their impact on human behavior is modulatory, with the main goal not being “happiness” but a “return to the most desirable state” – which can be highly context-dependent.

**Objectives:**

Review of the modulatory functions of endorphins on human behavior and their possible implications in psychiatric conditions.

**Methods:**

Pubmed search consisting of the MeSH terms “Endorphins”, “Opioid Peptides”, “Behavior”, and “Psychiatry”.

**Results:**

Endorphins elicit pleasure via stimulation of the release of dopamine from the ventral tegmental area to the *nucleus accumbens.* They are known to be involved in analgesia and stress response and social interaction. Endorphins can be released in a multitude of circumstances that may seem contradictory – having both inhibitory and stimulating roles in appetite, sexual response, and memory– but are modulatory effects depending on what constitutes homeostasis in each context. Peripheral levels of endorphins have been found low in depression and post-traumatic stress disorder. In schizophrenia, studies suggest that peripheral levels are high during psychosis, low in chronic disease and that naltrexone seems to improve auditory hallucinations. Endorphins may also have a role as markers of treatment response.

**Conclusions:**

Endorphins have a complex role in behavior and homeostasis. These molecules could have implications in psychiatry- given that they are part of our stress response and are released to promote a more “desirable state”. Their role as a marker of illness or response to treatment needs further investigation.

**Disclosure:**

No significant relationships.

